# Routes of Ca^2+^ Shuttling during Ca^2+^ Oscillations: FOCUS ON THE ROLE OF MITOCHONDRIAL Ca^2+^ HANDLING AND CYTOSOLIC Ca^2+^ BUFFERS*[Fn FN2]

**DOI:** 10.1074/jbc.M115.663179

**Published:** 2015-09-22

**Authors:** László Pecze, Walter Blum, Beat Schwaller

**Affiliations:** From Anatomy, Department of Medicine, University of Fribourg, Route Albert-Gockel 1, CH-1700 Fribourg, Switzerland

**Keywords:** calcium imaging, calcium intracellular release, calcium transport, calcium-binding protein, mitochondria, Ca^2+^ oscillations, calretinin, mitochondrial Ca^2+^ handling

## Abstract

In some cell types, Ca^2+^ oscillations are strictly dependent on Ca^2+^ influx across the plasma membrane, whereas in others, oscillations also persist in the absence of Ca^2+^ influx. We observed that, in primary mesothelial cells, the plasmalemmal Ca^2+^ influx played a pivotal role. However, when the Ca^2+^ transport across the plasma membrane by the “lanthanum insulation method” was blocked prior to the induction of the serum-induced Ca^2+^ oscillations, mitochondrial Ca^2+^ transport was found to be able to substitute for the plasmalemmal Ca^2+^ exchange function, thus rendering the oscillations independent of extracellular Ca^2+^. However, in a physiological situation, the Ca^2+^-buffering capacity of mitochondria was found not to be essential for Ca^2+^ oscillations. Moreover, brief spontaneous Ca^2+^ changes were observed in the mitochondrial Ca^2+^ concentration without apparent changes in the cytosolic Ca^2+^ concentration, indicating the presence of a mitochondrial autonomous Ca^2+^ signaling mechanism. In the presence of calretinin, a Ca^2+^-buffering protein, the amplitude of cytosolic spikes during oscillations was decreased, and the amount of Ca^2+^ ions taken up by mitochondria was reduced. Thus, the increased calretinin expression observed in mesothelioma cells and in certain colon cancer might be correlated to the increased resistance of these tumor cells to proapoptotic/pronecrotic signals. We identified and characterized (experimentally and by modeling) three Ca^2+^ shuttling pathways in primary mesothelial cells during Ca^2+^ oscillations: Ca^2+^ shuttled between (i) the endoplasmic reticulum (ER) and mitochondria, (ii) the ER and the extracellular space, and (iii) the ER and cytoplasmic Ca^2+^ buffers.

## Teaser

**Background:** Ca^2+^ oscillations in mesothelial cells depend on Ca^2+^ influx.

**Results:** However, the “lanthanum insulation method” renders the oscillations independent of extracellular Ca^2+^.

**Conclusion:** Multiple pathways of Ca^2+^ shuttling are operating simultaneously during Ca^2+^ oscillations.

**Significance:** Experimental and mathematical approaches shed light on the mechanism of Ca^2+^ oscillations.

## Introduction

The calcium ion (Ca^2+^) is a universal intracellular messenger that controls a diverse range of cellular processes including cell proliferation, apoptosis, fertilization, neurotransmitter release, and heartbeat among many others (1). Ca^2+^ pumps in the plasma membrane (plasma membrane Ca^2+^-ATPase) and in endoplasmic reticulum (ER)^2^ membranes (SERCA) are responsible for the low cytosolic (*c*_cyt_) and nuclear free Ca^2+^ concentrations (*c*_nucl_) (50–100 nm) compared with the free Ca^2+^ concentrations in the extracellular space (1–2 mm) and the ER lumen (*c*_ER_) (100–500 μm). At rest, the free Ca^2+^ concentration in the mitochondrial matrix (*c*_mito_) is close to the resting *c*_cyt_, but it rises to 20–30 μm during stimulation, *e.g.* in motor nerve terminals in *Drosophila melanogaster* (2). Cell activation in a wide range of cell types results in Ca^2+^ oscillations and in transient waves of increased *c*_cyt_ (3–6). These oscillations (or waves) are not restricted to *c*_cyt_, but also *c*_nucl_ (7), *c*_ER_ (8), and *c*_mito_ show Ca^2+^ oscillations (9). The spatial extent of the oscillatory Ca^2+^ signal is also important. (i) In astrocytes, the area of Ca^2+^ oscillations is sometimes restricted to only one protrusion regulating the release of gliotransmitters; *i.e.* different oscillatory frequencies can coexist at the same time within the same cell (10). (ii) In *Xenopus laevis* oocytes, regenerative spiral waves of release of free Ca^2+^ spread through the entire cell (11). (iii) Intercellular Ca^2+^ waves spreading via gap junctions occur in rat liver epithelial cells upon mechanical stimulation (12).

In cells maintained *in vitro*, serum starvation followed by readministration leads to intracellular Ca^2+^ signals, most often in the form of oscillations (13, 14). The precise mechanism(s) leading to these oscillations is poorly understood because serum contains a large number of known and as yet unidentified growth factors and mitogenic compounds, all potentially participating in this oscillatory activity (15). In Swiss 3T3 cells, serum-induced Ca^2+^ changes are essential but not sufficient to induce NF-κB activation and subsequent DNA synthesis (16). In some cell types, Ca^2+^ oscillations even persist in the absence of Ca^2+^ influx across the plasma membrane (3, 4), whereas in others, Ca^2+^ oscillations strictly depend on Ca^2+^ influx (5, 8). Mitochondria influence cytosolic Ca^2+^ oscillations in at least two ways. First, mitochondria produce ATP, which is required for SERCA and plasma membrane Ca^2+^-ATPase function, that results in Ca^2+^ extrusion and thus lowering of *c*_cyt_. Second, during *c*_cyt_ oscillations, *c*_mito_ also manifests oscillations, indicative of a role of mitochondria in shaping and/or modulating *c*_cyt_ oscillations (9). Ca^2+^ uptake into the mitochondria is determined by both the large negative voltage (−150 to −180 mV) across the inner membrane that results from the proton pumping by the respiratory chain and the Ca^2+^ concentration gradient between the cytoplasm and matrix (17). The mitochondrial calcium uniporter (MCU) is the key player responsible for the uptake of Ca^2+^ by mitochondria (18). The MCU has a rather low Ca^2+^ affinity and operates over a micromolar range of cytosolic Ca^2+^.

To address these questions, we performed lanthanum (La^3+^) insulation experiments where both the Ca^2+^ influx and efflux across the plasma membrane are blocked (19). We hypothesized that under these experimental conditions mitochondria serving as a Ca^2+^ store/source might substitute for this function normally exerted by the extracellular space. Using a genetically encoded Ca^2+^ indicator targeted to the mitochondria, we managed to verify this assumption *in vitro*. In addition, we investigated the effects of the following compounds on *c*_cyt_ oscillations and mitochondrial Ca^2+^ handling: the proton uncoupler carbonyl cyanide *m*-chlorophenylhydrazone (CCCP), the mitochondrial Na^+^/Ca^2+^ blocker CGP-37157, the mitochondrial MCU blocker Ru-360, and finally the “Ca^2+^-buffering” protein calretinin. Based on the experimental findings, we built a mathematical model for Ca^2+^ oscillations taking into account the various processes implicated in these oscillations.

## Materials and Methods

### 

#### 

##### Reagents

Thapsigargin, LaCl_3_, and EGTA were purchased from Sigma-Aldrich. CGP-37157 and BAPTA-AM were obtained from Tocris Bioscience (Bristol, UK). Ru-360 was purchased from Calbiochem, and Rhodamine 123 was from Invitrogen. EGTA-AM and tetramethylrhodamine methyl ester were purchased from AAT Bioquest (Sunnyvale, CA). CGP-37157 was dissolved in pure ethanol as 100 mm stock solutions. Thapsigargin and Rhodamine 123 were dissolved as 100 mm stock solutions in DMSO. BAPTA-AM and EGTA-AM were dissolved as 30 mm stock solutions in DMSO. LaCl_3_ was dissolved in double distilled water. The final concentrations of the solvents were <0.1% in all experimental solutions. At these concentrations, the solvents did not modify the evoked Ca^2+^ signals in control experiments (data not shown).

##### Isolation of Primary Mouse Mesothelial Cells

Primary mouse mesothelial cells (prMC) were isolated from 4–6-month-old C57Bl/6J mice according to an established protocol (20) and as applied in our previous study (8). The pelleted cells enriched in mouse mesothelial cells were grown in DMEM/F-12 GlutaMAX medium supplemented with 15% FCS; 0.4 μg/ml hydrocortisone; 10 ng/ml epidermal growth factor; 1% insulin, transferrin, and selenium; 1 mm sodium pyruvate; 0.1 mm β-mercaptoethanol; 1% non-essential amino acids; 1% penicillin-streptomycin; and 2% Mycokill (PAA Laboratories, Pasching, Austria) (21). After a few days (>4 days *in vitro*), cultured cells showed the typical cobblestone-like morphology of mesothelial cells, and cell cultures maintained for ∼60 days *in vitro* were used for the measurements.

##### Plasmids and Lentiviral Infection

For the generation of cell lines stably expressing the Ca^2+^ indicator proteins GCaMP3 (Addgene plasmid 22692 (22)) and mito-CAR-GECO1 (Addgene plasmid 46022 (23)), the lentiviral expression vector pLVTHM (Addgene plasmid 12247 (24)) was used. The GFP cassette in pLVTHM was replaced with cDNAs coding for the respective Ca^2+^ indicator proteins. Briefly, pGCaMP3 was produced in SCS110 dam^−^ bacteria and digested with AfeI and XbaI, and the fragment was inserted into the PmeI and SpeI sites of the backbone of pLVTHM to produce the final plasmid pLV-GCaMP3. The expression plasmid CMV-mito-CAR-GECO1 was used as template for the production of a DNA fragment coding for mito-CAR-GECO1. The required DNA fragment was synthesized by PCR using the following primers pairs: 5′-TAG CGT TTA AAC GGG CCC TC-3′ and 5′-GAG AAC TAG TTT ACT TCG CTG TCA TCA TTT GTA C-3′. The amplicon was digested with PmeI and SpeI and inserted into the unique sites of the pLVTHM vector to produce the final pLV-mito-CAR-GECO1 plasmid. Calretinin overexpression was achieved by the help of a lentiviral system. Briefly, the GFP cassette in pLVTHM was replaced with the human *CALB2* cDNA coding for full-length calretinin using the previously described expression plasmid RSV-CALB2-neo (25) as template. The DNA fragment coding for full-length calretinin was synthesized by PCR using the primers PmeI-CALB2 (5′-AGT CGT TTA AAC ATG GCT GGC CCG CAG CAG CAG-3′) and SpeI-CALB2 (AGT CAC TAG TTT ACA TGG GGG GCT CGC TGC A-3′). The amplicon was digested with PmeI and SpeI and inserted into the unique sites of the pLVTHM vector to produce the final pLV-CALB2 plasmid. We also generated a lentivirus expressing calretinin (CR) tagged with the enhanced blue fluorescent protein (EBFP) allowing for the easy identification of cells overexpressing EBFP-CR. For this, the pLV-EBFP2-nuc plasmid (Addgene plasmid 36085) and CMV-CALB2-neo were used. The DNA fragment coding for full-length calretinin was synthesized by PCR using the primers XhoI-CALB2 (5′-GAG ACT CGA GTA GCT GGC CCG CAG CAG C-5′) XbaI-CALB2 (5′-GAG ATC TAG ATT ACA TGG GGG GCT CGC TGC A-3′). The amplicon was digested with XhoI and XbaI and inserted into the unique sites of the pLV-EBFP2-nuc vector to produce the final pLV-EBFP2-CR plasmid. As a control plasmid coding for EBFP only (pLV-EBFP2-X), the nuclear localization signal was removed in the plasmid pLV-EBFP2-nuc by deleting an XhoI fragment. All lentiviral plasmids were verified by restriction enzyme digestion and sequencing. Lentivirus was produced by the calcium phosphate transfection method using HEK293T cells and three plasmids: one of the expression plasmids (*e.g.* pLV-GCaMP3 or pLV-mito-CAR-GECO1), the envelope plasmid (pMD2G-VSVG, Addgene plasmid 12259), and the packaging plasmid (psPAX2, Addgene plasmid 12260). Virus-containing supernatants were collected after 48 and 72 h, filtered, aliquoted, and frozen at −80 °C (26). MCU expression was knocked down in prMC expressing GCaMP3 and mito-CAR-GECO1 using Mission lentiviral transduction particles (Sigma-Aldrich) TRCN0000267404 and TRCN0000265169. Mission transduction particles directed toward human parvalbumin (TRCN000056549) and non-infected cells served as controls. Infected cells were selected using 2 μg/ml puromycin dihydrochloride (Sigma-Aldrich) for 1 week. *MCU* transcript knockdown was verified by qRT-PCR analysis.

##### qRT-PCR

PrMC were seeded in 6-well plates, and RNA was extracted with 1 ml of PeqGold Trifast (PeqLab, Erlangen, Germany). cDNA synthesis (QuantiTect Reverse Transcription kit, Qiagen, Hombrechtikon, Switzerland) and qRT-PCR (Rotor-Gene SYBR Green PCR kit, Qiagen) were performed following the manufacturers' protocols. Primers were as follows: m*UBC*: forward, 5′-GGA CGC CAC CGT GAA ACA ACT C-3′; reverse, 5′-ACC TCC AGG GTG ATG GTC TTA CCA-3′; m*MCU*: forward, 5′-CTC ACC AGA TGG CGT TCG AGT CG-3′; reverse, 5′-GCG TCG CTG CAT CTT CAT GGC T-3′.

##### Calcium Imaging

prMC were isolated as described before (27) and grown on collagen-coated glass bottom 35-mm dishes (MatTek Corp., Ashland, MA). The buffer solution (Hepes-buffered saline) used for Ca^2+^ imaging experiments contained 120 mm NaCl; 5.4 mm KCl, 0.8 mm Mg_2_Cl, 20 mm Hepes, 1 mm CaCl_2_, and 10 mm glucose, pH 7.4 (adjusted by NaOH). In the low Ca^2+^ solution, CaCl_2_ was replaced with an equimolar concentration of NaCl. The drugs (thapsigargin, FCS, and EGTA) were added to the solutions and remained in the solution until the end of the experiments. In some experiments, cells were pretreated either with 250 μm CGP-37157 or with 10 μm Ru-360 for 30 min at 37 °C. Cells were loaded either with 30 μm BAPTA-AM or 30 μm EGTA-AM for 15 min at 37 °C. We used a DMI6000 inverted confocal microscope integrated to a Leica TCS-SP5 work station to examine fluorescence signals indirectly, reporting *c*_cyt_ or *c*_mito_. The following excitation wavelengths were used to illuminate the fluorophores: 488 nm for GCaMP3 and 561 nm for mito-CAR-GECO1. Fluorescence emissions were recorded with a 20× objective and bandpass filters of 505–550 nm for GCaMP3 and 584–683 nm for mito-CAR-GECO1. Fluorescence images for *c*_cyt_ or *c*_mito_ measurements were collected every 3 s. Circle-shaped regions of interest (ROIs) were placed inside the cytoplasmic area of cells. The fluorescence values were calculated after background subtraction (fluorescence intensity of regions without cells). Fluorescence intensity values were normalized in each experiment to the averaged basal value preceding the treatment period. A bleaching correction was carried out when the baseline was not stable. LAS-AF (Leica) and Prism5 (GraphPad Software, Inc., San Diego, CA) software were used for data analysis.

##### ATP Measurements

PrMC were starved in serum-free DMEM supplemented with 1% penicillin-streptomycin for 24 h and distributed into 15 centrifuge tubes (50,000 cells/tube) in 50 μl of Hepes-buffered saline (+Ca^2+^). 1% FCS was added to 11 tubes, and 6 min later, 100 nm CCCP was added to five tubes. During the experiment, lysis buffer was added into each tube one after another with a delay of 1 min. ATP levels were determined using the ATP bioluminescence assay kit HS II according to the manufacturer's protocol (Roche Applied Science) with a microplate luminometer (PerkinElmer Life Sciences).

##### Mitochondrial Membrane Potential (ΔΨ) Measurements

Mito-CAR-GECO1-expressing prMC were seeded on glass bottom Petri dishes and incubated with 10 μm Rhodamine 123 for 20 min at room temperature. Cells were washed three times with Hepes-buffered saline (+Ca^2+^). During the recording using the confocal microscope, a 488-nm excitation wavelength was used to illuminate Rhodamine 123. Fluorescence emissions were recorded with a 20× objective and bandpass filters of 505–550 nm for Rhodamine 123. The distribution of Rhodamine 123 between the mitochondrial matrix and cytosol is proportional to the mitochondrial membrane potential. As the mitochondrial network is distributed within the entire cytoplasmic space, circle-shaped ROIs were randomly assigned to the cytoplasmic region for the fluorescence intensity measurements. The signal intensity is proportional to the amount of Rhodamine 123 dye incorporated by mitochondria in this ROI. For the normalization and thus the measurement of Rhodamine 123 released by mitochondria, an ROI within the nuclear region not containing mitochondria was selected, and the fluorescence intensity in this ROI was determined. The relative (rel.) ΔΨ was calculated according to the following equation.

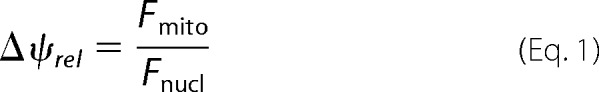
 where *F*_mito_ and *F*_nucl_ are the fluorescence intensity of Rhodamine 123 in the mitochondrial and nuclear regions, respectively. The mitochondrial membrane potential was additionally measured with tetramethylrhodamine methyl ester. For these measurements, cells were preincubated with 50 nm tetramethylrhodamine methyl ester for 30 min.

##### Estimation of the Intracellular Calretinin Concentration by Western Blot Analysis

Protein samples were isolated from cultured prMC. Cells were grown in 25-cm^2^ flasks and harvested at near confluence. Total proteins were extracted with ice-cold radioimmune precipitation assay buffer. Serial dilutions of protein extracts (50, 5, 0.5, and 0.005 μg) from each cell culture sample as well as 40 ng of purified human recombinant calretinin were loaded onto SDS-polyacrylamide gels (12.5%). After separation, proteins were transferred onto nitrocellulose membranes (Bio-Rad) and incubated overnight at 4 °C with the calretinin-specific antibody CR7699/4 (Swant, Marly, Switzerland) at a dilution of 1:10,000. Rabbit secondary antibody linked to horseradish peroxidase (Sigma-Aldrich) was diluted at 1:10,000, and membranes were incubated for 4 h. For the detection, the chemiluminescent reagent Luminata Classico Forte (EMD Millipore Corp., Billerica, MA) was used. Chemiluminescent and normal illumination digital images were recorded on a system from Cell Biosciences (Santa Clara, CA). Area densities of calretinin bands were measured with ImageJ software. From the density curves, the cell protein concentration corresponding to 40 ng of calretinin was determined. This allowed determination of calretinin or more precisely that of the fusion protein EBFP-calretinin content in μg/mg of total protein. Based on previous estimation of a protein concentration of about 0.2 g/ml (28) in mammalian cells, the intracellular concentration of EBFP-calretinin was estimated.

##### Frequency Determination and Amplitude Scan

Computerized peak recognition for frequency and amplitude analyses was realized via the Microsoft Excel 2010 environment as described before (8); normalized recordings from >30 oscillating prMC were evaluated. The oscillation frequency as well as the average amplitude was determined for three time windows: 1–5, 5–9, and 9–13 min after serum administration.

##### Mathematical Simulation

**FIGURE 1.**
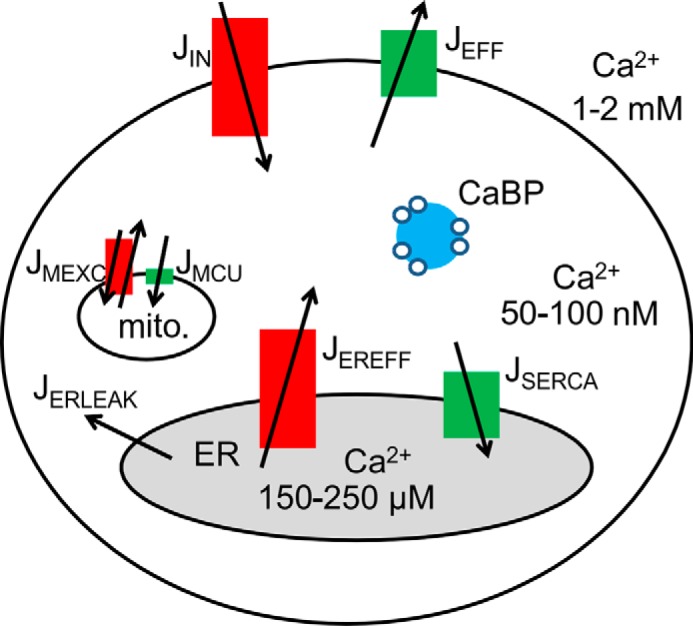
**Schematic model of cellular compartments and Ca^2+^ toolkit components implicated in Ca^2+^ oscillations in prMC.** The plasma membrane contains components responsible for Ca^2+^ influx (*J*_IN_) and efflux (*J*_EFF_). The dominant intracellular Ca^2+^ release (*J*_EREFF_) and uptake (*J*_SERCA_) systems are localized in ER membranes. A small constant leak (*J*_ERLEAK_) occurs independently of Ca^2+^ channels. The functions of the mitochondrial Ca^2+^ exchangers (Na^+^/Ca^2+^ exchanger and H^+^/Ca^2+^ exchanger) (*J*_MEXC_) and the mitochondrial calcium uniporter (*J*_MCU_) are responsible for the Ca^2+^ transport across the mitochondrial inner membrane; intracellular Ca^2+^ buffers (*CaBP*) such as calretinin acting as a transient cytosolic Ca^2+^ store/source modulate temporal aspects of *c*_cyt_ and consequently affect Ca^2+^ fluxes across all membranes (plasma membrane, ER, and mitochondria (*mito.*)).

To build the mathematical model, we considered four compartments: the extracellular space, cytoplasm, mitochondrial matrix, and ER lumen (Fig. 1). A fifth element placed within the cytoplasm in some simulations was the presence of a Ca^2+^ buffer. Membrane junctions between the ER and the plasma membrane ensured that the functional unit components (Ca^2+^ channels and pumps) are concentrated spatially in a very small space (29). Similarly close contacts were also assumed to exist between mitochondria and ER (30). One oscillatory unit represents an inositol trisphosphate receptor (InsP_3_R) cluster and its surrounding. We presumed that changes in *c*_cyt_, *c*_ER_, and *c*_mito_ of the entire cell were similar to that of individual units, *i.e.* spatially homogenous. In our view, this simplification is acceptable because the oscillations are slow and the cell size is small. In this case, the spatial diffusion of Ca^2+^ rapidly equilibrates the putative spatial differences and thus synchronizes the functions of individual functional units (31). In a cell with a 10-μm diameter, the diffusion is estimated to equilibrate spatial heterogeneity in *c*_cyt_ in less than 0.1 s (32). However, because Ca^2+^ waves not only depend on Ca^2+^ diffusion but also on the action of Ca^2+^ pumps and channels, the Ca^2+^ wave is ∼10 times slower (33). Although our model is a minimal deterministic point model and cannot produce the stochastic and spatial phenomena of the Ca^2+^ oscillations, it is a useful tool to illuminate the observed characteristics of the mitochondrial Ca^2+^ handling. Our aim was to build the most simple model still able to produce the experimentally observed phenomena.

Ca^2+^ transports across the plasma membrane were defined as *J*_IN_ and *J*_EFF_, and the transports across the ER membrane were termed *J*_SERCA_ and *J*_EREFF_, respectively. *J*_IN_ includes Ca^2+^ channels in the plasma membrane, *e.g.* voltage-gated Ca^2+^ channels, transient receptor potential channels, store-operated channels, P2X purinoreceptors, hyperpolarization-activated cyclic nucleotide-gated channels, etc. The *J*_EFF_ represents the pumps involved in Ca^2+^ extrusion, plasma membrane Ca^2+^-ATPases and Na^+^/Ca^2+^ exchangers. The SERCA pumps transport Ca^2+^ from the cytoplasm to the ER, whereas the *J*_EREFF_ represents the ER channels involved in emptying the ER, ryanodine receptor and InsP_3_R. The function of the mitochondrial exchangers (*J*_MEXC_) and the mitochondrial calcium uniporter (*J*_MCU_) are responsible for the Ca^2+^ transport across the mitochondrial inner membrane (see Fig. 1).

We denote by *c*_cyt_ the Ca^2+^ concentration (in nm) in the cytosol and by *c*_ER_ that in the lumen of the ER. Mitochondrial matrix free concentration is denoted by *c*_mito_. The equations for the model are as follows.

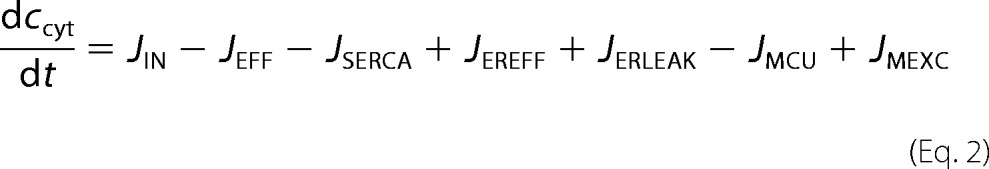


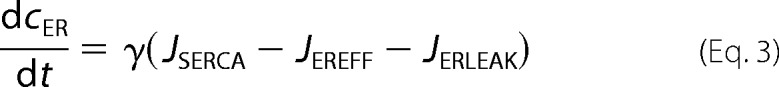


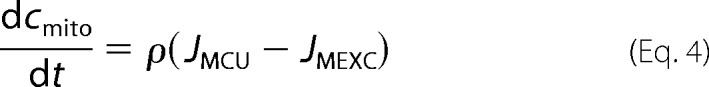
 where *J*_IN_ is the flux of Ca^2+^ ions entering the cell, *J*_EFF_ is the Ca^2+^ flux pumped out of the cell, *J*_SERCA_ denotes the Ca^2+^ flux pumped from the cytosol to ER, *J*_EREFF_ is the flux of Ca^2+^ passing from the ER to the cytosol, *J*_MCU_ denotes the function of MCU, *J*_MEXC_ displays the function of mitochondrial Ca^2+^ exchangers (mitochondrial Na^+^/Ca^2+^ and H^+^/Ca^2+^ exchangers), and finally *J*_ERLEAK_ represents a small flux of Ca^2+^ diffusing from the ER to the cytosol (all values in nm/s).

The constant γ is the ratio between the changes in *c*_cyt_ and *c*_ER_ caused by the same quantity of Ca^2+^ ions transported through the ER membrane. This value is derived from the difference in the effective volume of the ER lumen and the cytoplasm and from the different fraction of free and protein-bound Ca^2+^ in these compartments (34). The value of the γ parameter was estimated experimentally (8).

The quantity of Ca^2+^ pumped out of the cell through the plasma membrane increases as a function of the Ca^2+^ concentration in the cytosol. The Na^+^/Ca^2+^ exchangers have low Ca^2+^ affinity but high capacity for Ca^2+^ transport, whereas the plasma membrane Ca^2+^-ATPases have a high Ca^2+^ affinity but a low transport capacity. Although the individual components of extrusion systems are usually modeled by Hill equations (35), the overall flux can be simulated by a simple linear equation (36) based on the experimental results of Herrington *et al.* (37).

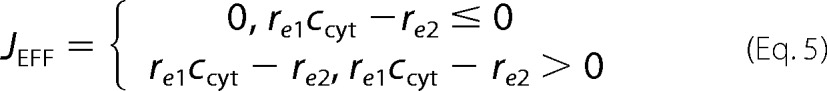
 where *r_e_*_1_ and *r_e_*_2_ are two positive constants.

SERCAs pump the Ca^2+^ ions from the cytosol to the ER. The quantity of the transported Ca^2+^ ions depends on *c*_cyt_ levels. We assume a linear relationship because the ER influx is also composed of different SERCA pumps with different *K_d_* values (38). Nevertheless, our model can also work when *J*_EFF_ and/or *J*_SERCA_ is simulated with the conventional Hill equations.

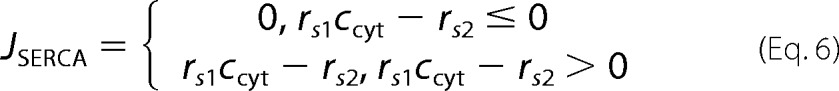
 where *r_s_*_1_ and *r_s_*_2_ are two positive constants.

Ca^2+^ ions are released from the ER to the cytosol through InsP_3_R and ryanodine receptor. Because we found experimentally that ryanodine receptor does not play a role in serum-induced oscillations in mesothelial cells (8) similarly to other non-excitable cells (39), we focused on InsP_3_R. In our model, InsP_3_R is influenced both by *c*_cyt_ and by *c*_ER_ but without an allosteric regulation between the two. InsP_3_R has Ca^2+^ binding sites not only on the cytoplasmic side but also on the luminal side (40). Experimental data show that an increase in inositol trisphosphate (InsP_3_) concentration causes a significant Ca^2+^ release from the ER in the absence of cytosolic Ca^2+^ (*c*_cyt_ = 0) (41). Moreover, the effects of luminal Ca^2+^ do not affect the cytosolic binding sites (42, 43). Therefore we modeled InsP_3_R function as the sum of two individual contributions.

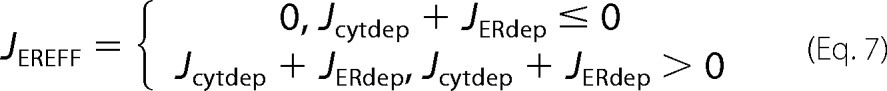
 where

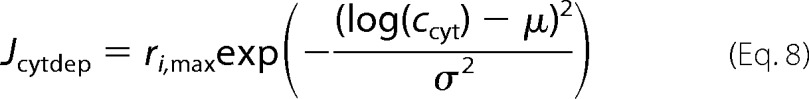
 and

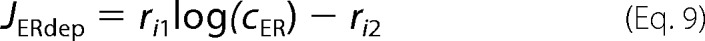
 with positive constants σ and *r_i_*_1_.

We introduced the dependence of InsP_3_R on the [InsP_3_], which has an influence both on *J*_cytdep_ and on *J*_ERdep_. According to the experimental data from several studies (44–46), elevating *c*_IP3_ mainly changes the mean and the maximum (μ and *r_i_*_,max_) of the bell-shaped curve of *c*_cyt_ dependence. Nevertheless, based on the experimental data presented (47, 48), elevating *c*_IP3_ also has an effect on the loading of the ER. Increased *c*_IP3_ reduces the amount of the stored Ca^2+^ ions. We simulated this effect by changing the *r_i_*_2_ parameter.

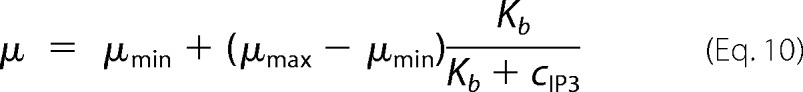


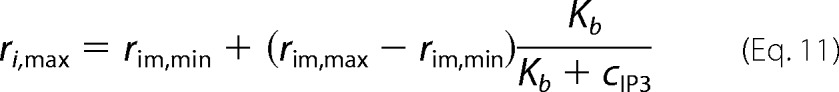


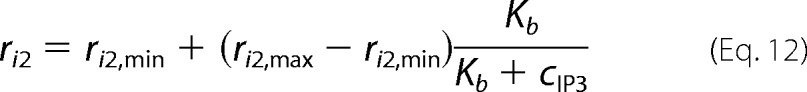
 where *K_b_* is the half-saturation constant of InsP_3_R for InsP_3_ and *c_IP3_* represents the InsP_3_R sensitivity to the inositol trisphosphate molecule, which was taken equal to inositol trisphosphate concentration in μm. μ_max_, μ_min_, *r*_im,min_, *r*_im,max_, *r_i_*_2,min_, and *r_i_*_2,max_ are positive constants. The parameter *J*_ERLEAK_ accounts for a Ca^2+^ flux from the ER to the cytoplasm independently of known Ca^2+^ channels, and this parameter is assumed to represent a small constant value (49).

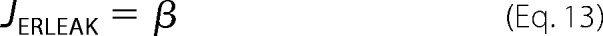


The outer membrane of mitochondria is freely permeable for Ca^2+^ ions, but the inner mitochondrial membrane provides a barrier. The constant ρ is the ratio between the changes in *c*_cyt_ and *c*_ER_ caused by the same quantity of Ca^2+^ ions transported through the mitochondrial inner membrane. This value is derived from the difference in the effective volume of the mitochondrial matrix and the cytoplasm and from the different fraction of free and protein-bound Ca^2+^ in these compartments.

There is a fast Ca^2+^ influx into the mitochondria matrix if *c*_cyt_ reaches a certain value. This fast influx is attributable to the function of MCU. We used Hill equations with a very high Hill coefficient as was done in the work of Marhl *et al.* (50). For simplicity, we did not take into account the changes in mitochondrial transmembrane potential and in mitochondrial volume during the Ca^2+^ oscillations in line with Marhl *et al.* (50), but we have to consider it during a protonophore treatment. The passage of calcium ions through the MCU requires the large membrane potential difference generated by the action of the electron transport chain (51).

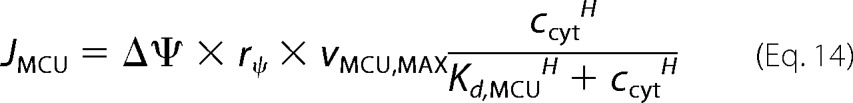
 where *r*_Ψ_ and *v*_MCU,max_ are positive constants, *K_d_*_,MCU_ is the dissociation constant of MCU for Ca^2+^ ions, and *H* is the Hill coefficient. In our model, *J*_MCU_ has a constant basal activity. That ensures that mitochondria can store a small amount of Ca^2+^ ions, which are released into the cytoplasm immediately after the collapse of the mitochondrial membrane potential. Higher ΔΨ means increased Ca^2+^ uptake but slower mitochondrial Ca^2+^ release.

To simulate the function of mitochondrial exchangers (Na^+^/Ca^2+^ and H^+^/Ca^2+^ exchangers), we consider that both will transport Ca^2+^ ions with a low velocity when there is a concentration gradient between the two sides of the mitochondrial inner membrane. For the simplicity, we neglected the changes in sodium and proton concentrations during the Ca^2+^ oscillations. Depending on the calcium concentration gradient, the exchangers can work in both directions.

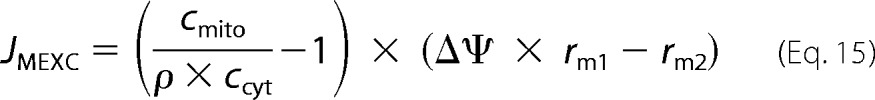
 where *r_m_*_1_ and *r_m_*_2_ are positive constants.

The Ca^2+^ influx across the plasma membrane is composed of passive leakage and the agonist-activated fluxes: the capacitive (store-operated channel-dependent) and the non-capacitive (arachidonate- or diacylglycerol-regulated) Ca^2+^ influx (52). We simulated the changes in *J*_IN_ starting from the beginning of the administration of serum (*t*_1_) using the following equations.

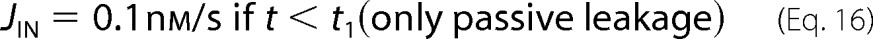


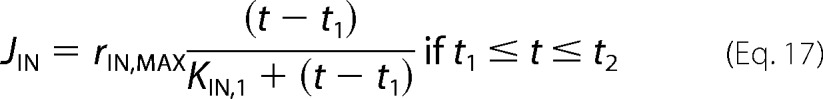


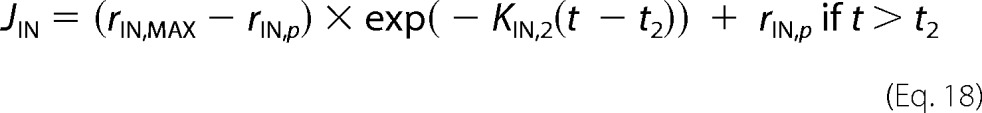


We simulated the changes in *c*_IP3_ from the beginning of the administration of serum (*t*_1_) with the following equations. The resting *c*_IP3_ was set to 15 nm (53).

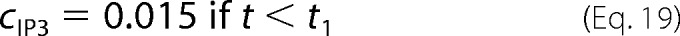


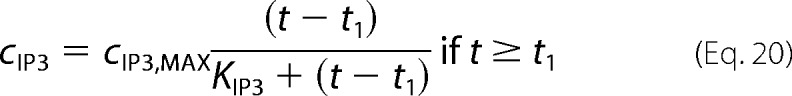


To simulate the effect of calretinin, we neglected its fast kinetics. Because this protein is considered as a fast Ca^2+^ buffer (54), calretinin reaches the Ca^2+^ steady state in a few milliseconds, which is much faster than our observed Ca^2+^ changes lasting for a few seconds. The fast kinetics of calretinin plays an important role at the mouth of voltage-gated Ca^2+^ channels in excitable cells (55) where fast and large changes in Ca^2+^ concentrations are expected.

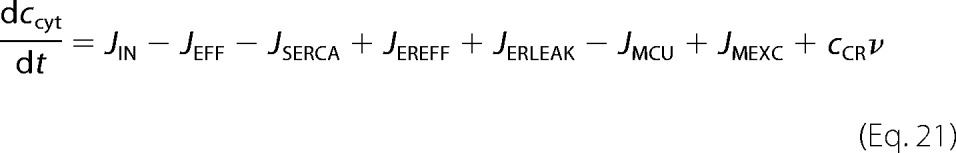
 where *c*_CR_ is the concentration of calretinin in the cytoplasm and ν is the average number of the Ca^2+^ binding sites of calretinin occupied by Ca^2+^. Calretinin has four high affinity Ca^2+^ binding sites and one low affinity binding site. The binding kinetics of the Ca^2+^ binding sites were simulated with Hill equations.

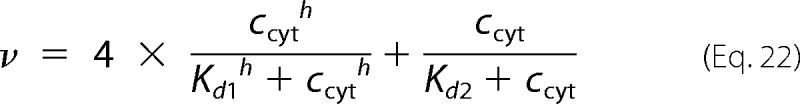
 where *K_d_*_1_ is the dissociation constant for the high affinity Ca^2+^ binding sites, *K_d_*_2_ is the dissociation constant for low affinity Ca^2+^ binding site, and *h* is the Hill coefficient for the high affinity binding sites. Among the high affinity Ca^2+^ binding sites, there is a positive cooperativity (*h* > 1). The values for the parameters came from the study of Faas *et al.* (54).

The values of each parameter are listed in Table 1. The initial values of parameters are derived either from our experiments in primary mesothelial cells or from fitting to experimental data previously reported in the above mentioned articles. The presented values are the result of the sequential fitting of the initial values to our *in situ* recordings. All computations of the model were implemented in the Microsoft Excel 2010 environment. The model system was discretized with a temporal resolution of 0.1 s (supplemental Excel document). There were no significant differences in the solution of the differential equations if we increased the temporal resolution (not shown). For visualization, Prism5 (GraphPad Software, Inc.) software was used.

**TABLE 1:** **Parameters used for the modeling**

Equation to determine	Parameter name	Value
	γ	450
	ρ	4
*J*_EFF_ (Equation 5)*^a^*	*r_e_*_1_	0.17/s
	*r_e_*_2_	18.8 nm/s
*J*_SERCA_ (Equation 6)*^a^*	*r_s_*_1_	0.27/s
	*r_s_*_2_	22 nm/s
*J*_EREFF_ (Equations 8 and 9)	σ	0.14142 nm
	*r_i_*_1_	1300/s
μ (Equation 10)	μ_min_	2.4 nm
	μ_max_	2.18 nm
	*K_b_*	2 μm
*r_i_*_,max_ (Equation 11)	*r*_im,min_	821.3 nm/s
	*r*_im,max_	24.3 nm/s
	*K_b_*	2 μm
*r_i_*_2_ (Equation 12)	*r_i_*_2,min_	6352 nm/s
	*r_i_*_2,max_	7042 nm/s
	*K_b_*	2 μm
*J*_ERLEAK_ (Equation 13)	β	2.5 nm/s
	*c*_ER_ (initial)	260 μm
	*c*_cyt_ (initial)	110 nm
	ΔΨ (initial)	180 mV
*J*_MCU_ (Equation 14)	*r*_Ψ_	0.005555/mV
	*v*_MCU,MAX_	5 nm/s
	*K_d_*_,MCU_	208 nm
	*H*	7.4
*J*_MEXC_ (Equation 15)	*r_m_*_1_	−0.01251/mV
	*r_m_*_2_	2.295
ν (Equation 22)	*K_d_*_1_	2.5 μm
	*K_d_*_2_	53 μm
	*h*	2.4
*J*_IN_ (Equations 16, 17, and 18)	*t*_1_	60 s
	*t*_2_	72 s
	*r*_IN,MAX_	5 nm/s
	*K*_IN,1_	0.01 s
	*r*_IN,_*_p_*	1.05 nm/s
	*K*_IN,2_	2 s
*c*_IP3_ (Equations 19 and 20)	*c*_IP3,MAX_	1.8 μm
	*K*_IP3_	0.1 μm

*^a^* Alternatively, the *J*_EFF_ and *J*_SERCA_ can be simulated conventionally with a Hill equation with the following parameters: *V*_max_, 260 and 170 nm/s; *K_d_*, 460 and 480 nm; and Hill coefficients, 3.5 and 2.4, respectively.

## Results

### 

#### 

##### Characterizing Ca^2+^ Fluctuations in Mitochondria of Primary Mouse Mesothelial Cells

**FIGURE 2.**
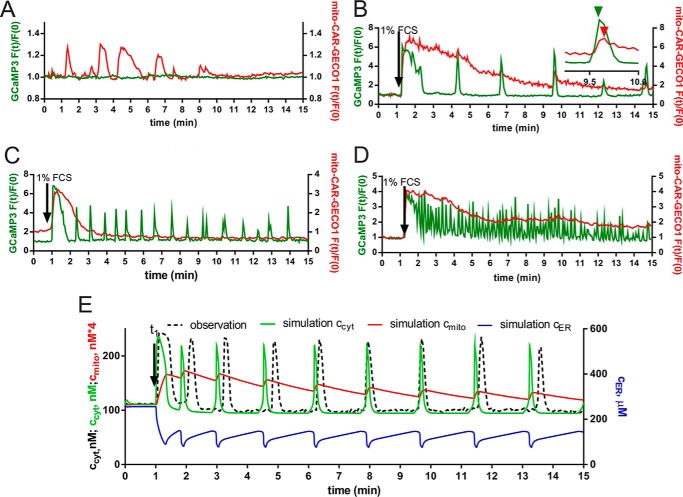
**Ca^2+^ oscillations in *c*_cyt_, *c*_ER_, and *c*_mito_ in prMC.** *A*, a representative recording shows spontaneous mitochondrial Ca^2+^ transients (*red* trace) in a resting cell without changes in *c*_cyt_ (*green* trace). *B–D*, single cell fluorescence recordings derived from time lapse videos show the simultaneous changes in *c*_cyt_ (*green* traces) and *c*_mito_ (*red* traces) after serum readministration. Despite the different oscillatory frequencies (increasing from *B* to *D*) in *c*_cyt_, the traces for *c*_mito_ were very similar in all three recordings: a fast initial rise followed by a quasiexponential decay with varying kinetics. The small *inset* in *A* shows (at an expanded time scale) that small fluctuations (short rises in *c*_mito_) coincide with *c*_cyt_ spikes. The peak in *c*_mito_ is slightly delayed in comparison with the peak in *c*_cyt_. *E*, an experimental recording (*black dashed line*) in prMC showing low frequency oscillations was selected for the fitting. The model shows the changes in *c*_cyt_ (*green* trace), *c*_mito_ (*red* trace), and *c*_ER_ (*blue* trace). The parameters and equations are presented in the supplemental Excel document. The changes in *c*_mito_ consist of an initial rise after FCS administration followed by a slow return to basal levels. Each Ca^2+^ spike in *c*_cyt_ results in a small hump in *c*_mito_ reaching its relative maximum with a small delay compared with the maximum in *c*_cyt_. Original recordings for *c*_ER_ in prMC were previously presented (8); they show sawtooth-like waves at a semidepleted ER state during Ca^2+^ oscillations as shown in our model here (*blue* trace).

In the absence of serum, prMC did not show Ca^2+^ oscillations as reported before (8). However, in a small fraction of cells (2–3%), isolated arrhythmic mitochondrial increases in *c*_mito_ were present without detectable changes in *c*_cyt_ (Fig. 2*A*). The addition of 1% FCS to the cell culture medium containing prMC that were grown in the absence of serum for 24 h resulted in a sudden rise of *c*_cyt_ lasting, on average, for ∼40 s followed by Ca^2+^ oscillations (Fig. 2, *B–D*). The percentage of prMC responding to serum readministration with Ca^2+^ oscillations was in the order of 70%. Non-oscillatory cells showed only an initial single Ca^2+^ transient or a so-called peak-plateau response (53). A wide range of different oscillatory patterns in *c*_cyt_ was present in a supposedly homogenous population of mesothelial cells. Most cells displayed long period (>10 min) baseline spiking oscillations with various frequencies of one spike per 3 min (Fig. 2*B*) up to 10 per min (Fig. 2*D*); also maximal spike amplitudes varied between individual cells. The baseline spiking oscillations represent discrete Ca^2+^ transients starting from a constant basal *c*_cyt_ level (Fig. 2, *B* and *C*). Sinusoidal oscillation is a term for a continuous fluctuation in *c*_cyt_ starting from a *c*_cyt_ value that is higher than the resting (basal) *c*_cyt_ (Fig. 2*D*). Most probably, sinusoidal oscillations are the result of high frequency overlapping baseline spiking oscillations (56). In prMC maintained in cell culture medium for longer periods (>10 passages), the percentage of the cells showing sinusoidal oscillations was increased as exemplified in Fig. 2*D*. However, the percentage of cells showing oscillatory activity was rather low at higher passages (∼20–40% at passages >10). The average frequency of the baseline spiking oscillations was found to be 15, 13, and 13 mHz in the following time segments: 1–5, 5–9, and 9–13 min after serum administration, respectively. The average amplitude of spikes (-fold increase in GCaMP3 fluorescence intensity) was found to be 2.66, 2.53, and 2.53 in the above mentioned time segments. By using two Ca^2+^ indicators targeted to either the mitochondrial matrix (mito-CAR-GECO1) or the cytoplasm (GCaMP3), we simultaneously monitored changes in *c*_mito_ and *c*_cyt_, respectively. The initial serum-induced rise in *c*_cyt_ was paralleled by a rapid rise in *c*_mito_ that reached the peak value within 30 s after the addition of 1% FCS (Fig. 2, *B–D*); from then on, *c*_mito_ decreased continuously until it reached its initial basal value, generally within the time span of 15 min monitored in most experiments. High frequency oscillations in *c*_cyt_ resulted in continuous elevation in *c*_mito_ (Fig. 2*D*). The rate of decay in *c*_mito_ was also rather variable between cells, and oscillations in *c*_cyt_ did not stop when *c*_mito_ had reached its basal levels. In some cells during the decreasing phase in *c*_mito_, small fluctuations (short rises in *c*_mito_) coincided with the *c*_cyt_ spikes but with a small delay (*e.g.* shown in Fig. 2*B*, *inset*). For the modeling, we took into account our previous results where basal and maximal *c*_cyt_ values during Ca^2+^ spikes in prMC were found to be 100 and 200–300 nm, respectively (8). Similarly, the values for the resting *c*_ER_ were taken as 150–250 μm, and the values after serum readministration were taken as 100–150 μm (8). The pattern of *c*_ER_ changes is best described as a sawtooth wave (8). These data were incorporated to build the mathematical model where Ca^2+^ concentrations in all compartments (*c*_cyt_, *c*_mito_, and *c*_ER_) were calculated and fitted to one *c*_cyt_ recording (Fig. 2*E*). The model accurately recapitulated the experimental findings, in particular with respect to *c*_mito_, which had not been modeled in our previous study (8). The pattern in *c*_mito_ is best described as a sudden rise after serum readministration followed by a rather smooth decay phase with small humps (increases in *c*_mito_) as the result of the oscillatory Ca^2+^ spikes.

##### Modulation of Mitochondrial Ca^2+^ Transport Mechanisms Affects Ca^2+^ Oscillations

**FIGURE 3.**
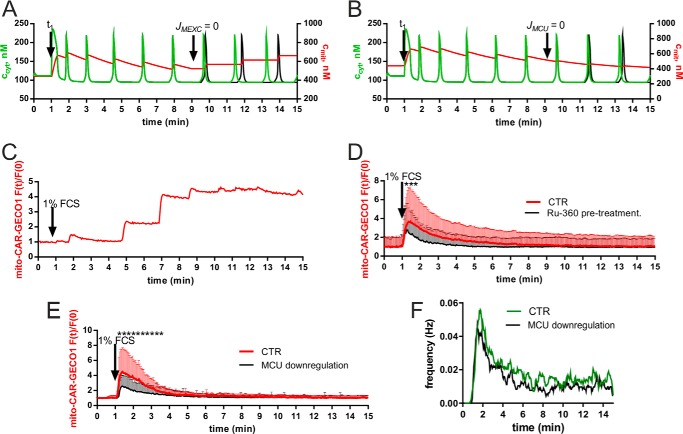
**Modulation of mitochondrial Ca^2+^ transport in prMC.** *A*, in the simulation, blocking of the mitochondrial Na^+^/Ca^2+^ exchanger activity (*J*_MEXC_ = 0) at 9 min predicts a staircase-like increase in *c*_mito_ (*red* trace) following each Ca^2+^ spike, and the oscillation frequency is expected to decrease (*black* trace) compared with the unperturbed situation (*green* trace). *B*, in the simulation, MCU activity was stopped (*J*_MCU_ = 0) at 9 min. This resulted in the absence of humps in *c*_mito_ following the Ca^2+^ spikes and a decrease in oscillation frequency (compare *green* trace for *c*_cyt_ (control conditions) with *black* trace) when *J*_MCU_ = 0. *C*, a representative recording of *c*_mito_, obtained in prMC pretreated for 30 min with CGP-37157 (50 μm), which blocked mitochondrial Ca^2+^ release, is depicted. As predicted by the model (*A*), a staircase-like increase in *c*_mito_ was observed after each cytosolic Ca^2+^ spike. *D*, reduction in mitochondrial Ca^2+^ uptake was observed in oscillatory prMC pretreated with 10 μm Ru-360; data are mean + S.D. from 10 cells each. *Asterisks* indicate significance at *p* < 0.05. *E*, reduction in mitochondrial Ca^2+^ uptake was observed in oscillatory prMC with down-regulated MCU; data are mean + S.D. from 20 cells each. *Asterisks* indicate significance at *p* < 0.05. *F*, frequency scan analyses of control (*CTR*) cells (*green* trace) and shMCU-treated (*black* trace) cells (*n* ≥ 50 oscillating cells for both conditions). In all time windows, the frequency was lower in cells where MCU had been down-regulated. *Error bars* represent S.D.

In the next series of experiments and modeling simulations, we investigated how altering mitochondrial function, mostly in relation to Ca^2+^ handling, affects *c*_cyt_ oscillations. The simulation showed that inhibition of the mitochondrial Ca^2+^ release (*J*_MEXC_) or mitochondrial Ca^2+^ uptake (*J*_MCU_) during Ca^2+^ oscillations decreased the oscillation frequency (Fig. 3, *A* and *B*). The experimental verification of our predictions was hampered by the absence of pharmacological mitochondrially targeted compounds that immediately reach the mitochondrial inner membrane when added to the recording solution. Briefly, after serum readministration, the addition of CGP-37157 (50 μm), a nonspecific blocker of the mitochondrial Na^+^/Ca^2+^ exchanger, had no effect on the patterns of Ca^2+^ oscillations in either *c*_cyt_ or *c*_mito_ (data not shown). However, some cells pretreated with CGP-37157 for 30 min displayed stairlike increases in *c*_mito_ (Fig. 3*C*), an effect predicted from our model (see Fig. 3*A*, *right part* (*c*_mito_)). Ruthenium compounds, *e.g.* ruthenium red and Ru-360, are potent and effective blockers of MCU in isolated mitochondria, but their usefulness for intact cells is limited by their poor membrane permeability and selectivity (57). Pretreatment of cells with 10 μm Ru-360 reduced the average oscillation frequency (approximately a 30% decrease during each time segment) and the initial mitochondrial Ca^2+^ uptake (Fig. 3*D*). However, it is currently still unclear whether in intact cells Ru-360 acts uniquely by the inhibition of the mitochondrial Ca^2+^ uptake or additionally by the inhibition of the extracellular Ca^2+^ influx. Therefore, we also used a molecular approach, *i.e.* down-regulation of MCU by shRNA to decrease the mitochondrial Ca^2+^ uptake. The down-regulation of *MCU* mRNA levels by 60–90% as determined by qRT-PCR resulted in a 40–60% decrease in the initial mitochondrial Ca^2+^ uptake (Fig. 3*E*). This led to an ∼20% reduction in the *c*_cyt_ oscillation frequency calculated by frequency scan analysis (Fig. 3*F*) in line with the predictions from our model (Fig. 3*B*). In all time windows (bins of 3 s), the oscillation frequency was lower in prMC where MCU had been down-regulated. Thus, both approaches (Ru-360 and shMCU) underscore the importance of mitochondria in Ca^2+^ oscillations.

**FIGURE 4.**
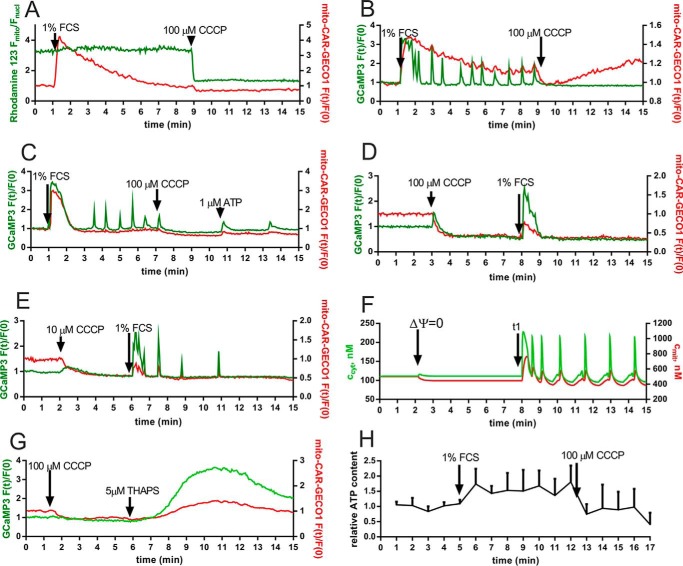
**Effect of the proton uncoupler CCCP on Ca^2+^ oscillations.** *A*, serum addition to prMC led to a rapid rise in *c*_mito_ (*red* trace) followed by a gradual decay. Addition of CCCP (100 μm) resulted in a rapid collapse of the membrane potential as evidenced by measuring Rhodamine 123 fluorescence signals (*green* trace). As the result of CCCP application, *c*_mito_ immediately returned to basal levels because the slightly elevated *c*_mito_ level (compared with the *c*_cyt_ level) could not be maintained when the driving force for mitochondrial Ca^2+^ uptake was eliminated. *B–H*, the role of the membrane potential and ATP treatment was investigated in greater detail, and one representative recording for each experimental condition is depicted. *B*, the addition of CCCP (100 μm) at *t* = 9 min blocked the serum-induced cytosolic Ca^2+^ oscillations. CCCP treatment led to a rapid fall in *c*_mito_; in some cells, a slow increase in *c*_mito_ occurred afterward (*B*), whereas in others, *c*_mito_ remained low (not shown). *C*, administration of ATP (1 μm) reestablished the CCCP-inhibited Ca^2+^ oscillations in some cells. *D*, addition of CCCP (100 μm) prior to serum readministration lowered the resting *c*_mito_ (also reflected by the simultaneous small increase in *c*_cyt_). However, from then onward, *c*_cyt_ dropped to levels lower then the basal *c*_cyt_ before CCCP treatment. The serum readministration at *t* = 8 min evoked only a single Ca^2+^ transient. *E*, at a lower CCCP concentration (10 μm), addition of 1% FCS at *t* = 6 min resulted in elevations in *c*_cyt_ and *c*_mito_ followed by a few oscillations in *c*_cyt_. The small amount of Ca^2+^ ions taken up by mitochondria (*red* trace) during Ca^2+^ spikes (*green* trace) was released back to the cytosol almost immediately. *F*, in this simulation experiment, the mitochondrial membrane potential was switched off (ΔΨ = 0) at *t* = 3 min; this resulted in a slight increase in *c*_cyt_ and a decrease in *c*_mito_ to values lower than basal *c*_mito_ as observed experimentally in *D* and *E*. Serum readministration evoked oscillatory activity in *c*_cyt_ and *c*_mito_. *G*, after the CCCP-induced collapse in ΔΨ, ER Ca^2+^ release by thapsigargin (5 μm) resulted in mitochondrial Ca^2+^ uptake (*red* trace) independent of ΔΨ. A representative recording displays the simultaneous changes in *c*_mito_ (*red*) and *c*_cyt_ (*green*). *H*, during FCS-induced Ca^2+^ oscillations, ATP levels in prMC were increased but dropped quickly after CCCP administration. The panel shows the mean + S.D. of three independent experiments. *Error bars* represent S.D.

CCCP is an inhibitor of oxidative phosphorylation by acting as a protonophore; *i.e.* it allows H^+^ to cross the inner mitochondrial membrane, resulting in the collapse of ΔΨ. During Ca^2+^ oscillations, ΔΨ was slightly increased (more negative), but it collapsed immediately after CCCP (100 μm) treatment (Fig. 4*A*). The collapse of the membrane potential after addition of 10 or 100 μm CCCP was also confirmed by using the tetramethylrhodamine methyl ester indicator dye (data not shown). When applied during Ca^2+^ oscillations resulting from serum readministration, CCCP blocked Ca^2+^ oscillations at 100 μm but not at 10 μm (data not shown). An immediate drop in *c*_mito_ was observed after CCCP treatment (Fig. 4*B*) followed by a continuous elevation in *c*_mito_ in some cells (∼20%) but not in others (Fig. 4, *A* and *C*). In a few cases (∼5% of cells), administration of ATP (1 μm) partially reverted the CCCP-induced oscillation stop (Fig. 4*C*). However, addition of ATP to the recording solution in the absence of serum was equally able to evoke Ca^2+^ oscillations in some cells (data not shown). The reason for this effect is currently unknown; ATP might act on receptors on the surface of prMC but was also shown to cross the plasma membrane and to have an impact from the intracellular side (58). Application of CCCP before serum administration led to an immediate fall in the basal *c*_mito_, reaching a new plateau 1–2 min later; the fall in *c*_mito_ was accompanied by a visible small rise in *c*_cyt_ in ∼20% of cells, indicative of a release of mitochondrial Ca^2+^ to the cytosolic compartment. In addition, CCCP also decreased the basal level in *c*_cyt_ (both at 100 and 10 μm), signifying that also the plasma membrane potential was affected. Serum readministration following CCCP (100 μm) treatment was still able to briefly elevate both *c*_cyt_ and *c*_mito_, but the increase in *c*_mito_ was smaller compared with cells not treated with CCCP; moreover, *c*_mito_ returned quickly to the level reached after CCCP addition, *i.e.* not to basal levels before treatment (Fig. 4*D*). Serum readministration after treatment with the lower CCCP concentration (10 μm) evoked low amplitude Ca^2+^ oscillations, and the mitochondrial Ca^2+^ rise during a cytosolic Ca^2+^ spike was small, and *c*_mito_ immediately returned to levels before serum administration but to lower levels than the basal *c*_mito_ before CCCP administration (Fig. 4*E*). The model also correctly predicted that the collapse in ΔΨ (at *t* = 3 min) resulted in a lower *c*_mito_. Serum administration (modeled as increasing *J*_IN_ and *c*_IP3_) led to an increase in *c*_cyt_ and *c*_mito_ followed by oscillations in *c*_cyt_ and *c*_mito_ (Fig. 4, compare *F* with the experimental recording shown in *E*). To provide more evidence for the presence of ΔΨ-independent mitochondrial Ca^2+^ uptake as shown in Fig. 4*D*, we induced Ca^2+^ release from the ER by thapsigargin after CCCP administration (Fig. 4*G*). We observed a rise not only as expected in *c*_cyt_ but in parallel also in *c*_mito_, confirming the existence of a ΔΨ-independent mitochondrial Ca^2+^ uptake. Addition of 1% FCS also resulted in an increase in the intracellular ATP concentration that lasted during the entire period of Ca^2+^ oscillations. Shortly after the collapse of ΔΨ induced by CCCP, an immediate fall in ATP levels was observed (Fig. 4*H*). Overall, our findings indicate that the oscillation stop induced by the protonophore CCCP is not exclusively the result of the decreased mitochondrial Ca^2+^ uptake but also mediated via CCCP-induced changes in plasmalemmal Ca^2+^ influx and decreased ATP production.

##### The Role of Ca^2+^ Influx on Ca^2+^ Oscillations and on Mitochondrial Ca^2+^ Handling

**FIGURE 5.**
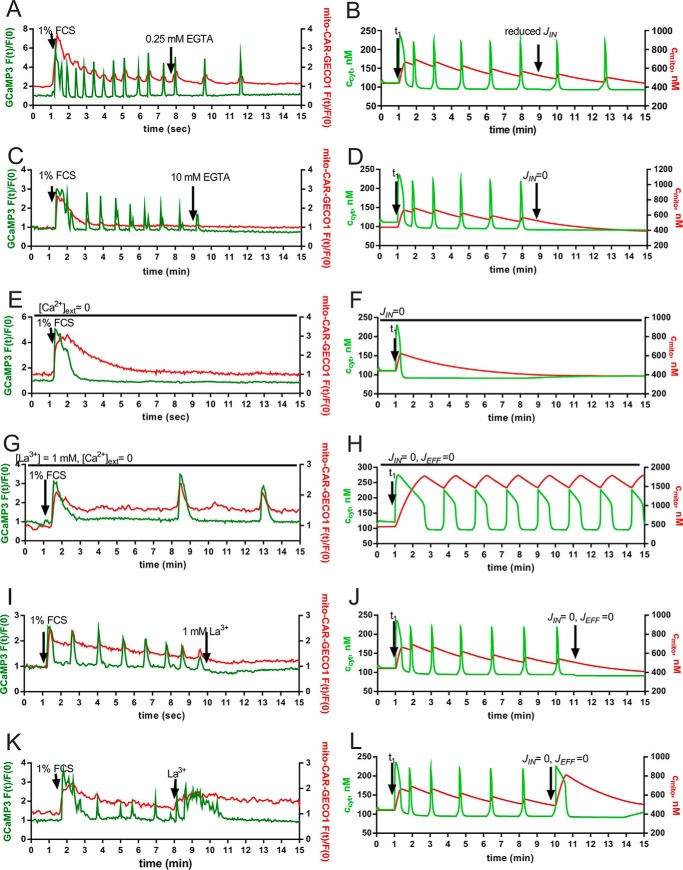
**Modulation of Ca^2+^ transport across the plasma membrane: representative experimental findings (*left panels*) and model simulations (*right panels*).** *A*, a decrease in external [Ca^2+^] from 1 to ∼0.75 mm by administration of 0.25 mm EGTA results in a decreased oscillation frequency. *B*, the model was able to recapitulate the effect of reduction of Ca^2+^ influx on Ca^2+^ oscillations. The parameter *r*_IN,_*_p_* representing *J*_IN_ was reduced from 0.85 to 0.6 nm/s at *t* = 9 min. *C*, removal of extracellular Ca^2+^ by the addition of EGTA (10 mm) at *t* = 9 min resulted in one final Ca^2+^ spike before cessation of oscillations. *D*, the model correctly predicted an immediate stop in Ca^2+^ oscillations when *J*_IN_ was decreased to zero. *E*, in the absence of extracellular Ca^2+^ ([Ca^2+^]*_o_* < 1 μm), prMC did not show Ca^2+^ oscillations. In a few cells, a single Ca^2+^ transient was visible. *F*, model traces for *c*_cyt_ (*green*) and *c*_mito_ (*red*) in “zero Ca^2+^” (*J*_IN_ = 0) confirmed the experimental findings shown in *E. G*, addition of Lanthanum chloride (La^3+^ insulation) prior to serum administration rendered Ca^2+^ oscillations independent of extracellular Ca^2+^ ions. Note that (i) *c*_mito_ remained elevated during the entire period (no slow decay phase), (ii) the frequency of Ca^2+^ oscillations was lower than in control conditions (*e.g.* as shown in the initial period in *A* or *C*), and (iii) the half-width of Ca^2+^ transients representing the duration of a Ca^2+^ spike was increased. *H*, the model confirmed that mitochondria were able to substitute for the role of the extracellular Ca^2+^ reservoir during Ca^2+^ oscillations. In line with the experimental findings, the half-width of Ca^2+^ spikes was increased, and *c*_mito_ remained elevated. *I–K*, La^3+^ insulation induced after serum administration (at *t* ≈ 8–10 min) blocked the Ca^2+^ oscillations either with (*K*) or without (*I*) a final large Ca^2+^ transient. *J–L*, the model was able to recapitulate both phenomena: it revealed that *c*_TOTAL_, *i.e.* the total amount of Ca^2+^ ions in the cell (*c*_mito_ + *c*_cyt_ + *c*_ER_), determined the response to La^3+^ insulation.

A decrease in extracellular [Ca^2+^] by the addition of 0.25 mm EGTA to the extracellular solution resulted in a reduction in the oscillation frequency (Fig. 5*A*). In this condition, *i.e.* when *c*_cyt_ oscillations were not blocked completely, the amplitude of the Ca^2+^ signals was not affected, and the pattern of mitochondrial Ca^2+^ release/uptake was not affected (Fig. 5*A*). This could be accurately modeled in our simulation (Fig. 5*B*). When oscillations were induced by the addition of 1% FCS to the Ca^2+^-containing recording solutions ([Ca^2+^]*_o_* ≈ 1 mm), decreasing [Ca^2+^]*_o_* to <1 μm by the addition of 10 mm EGTA at *t* = 9 min resulted in an immediate stop of the oscillations, indicating the necessity of Ca^2+^ influx for the oscillations in *c*_cyt_ (Fig. 5*C*). Removal of the extracellular Ca^2+^ had no visible effect on the decay curve of *c*_mito_, and basal levels were reached at the end of the observation period (15 min). When the serum readministration was carried out in the “Ca^2+^-free” condition, most prMC did not show any response in *c*_cyt_. In ∼5% of prMC, an initial small rise in *c*_cyt_ was observed but without signs of Ca^2+^ oscillations in support of the hypothesis that extracellular Ca^2+^ is essential for the sustained oscillations (Fig. 5*E*). Interestingly, different results were obtained in the Ca^2+^-free condition when both Ca^2+^ influx and efflux across the plasma membrane were blocked by the addition of 1 mm La^3+^, the so-called lanthanum insulation (19, 59), prior to the serum readministration. After serum addition, an immediate rise in *c*_cyt_ and *c*_mito_ was detected; although *c*_cyt_ decayed to basal levels within the next 2 min, *c*_mito_ remained elevated and did not show the typical decay curve as seen *e.g.* in Fig. 5*A*, *C*, and *E*. Moreover, long lasting but slow oscillations in *c*_cyt_ were observable (Fig. 5*G*), and at each cytosolic Ca^2+^ spike, a corresponding spike in *c*_mito_ occurred. This indicates that during the La^3+^ insulation a considerable amount of Ca^2+^ ions released from the ER, leading to the transient increase in *c*_cyt_, is taken up by mitochondria as evidenced by the mitochondrial Ca^2+^ spikes (Fig. 5*G*). Thus, blocking the Ca^2+^ efflux across the plasma membrane leads to a shuttling of the Ca^2+^ ions between the ER and mitochondria, leading to these slow oscillations. Of note, the mitochondria remain in a rather Ca^2+^-loaded state because Ca^2+^ cannot be transported out of the cell. We estimate that the mitochondrial Ca^2+^ uptake and release velocities likely determine the frequency of Ca^2+^ oscillations. La^3+^-induced blocking of the Ca^2+^ transport across the plasma membrane at a time point when serum-induced Ca^2+^ oscillations were ongoing led to a complete block of the oscillations (Fig. 5, *I* and *K*). In some cases, La^3+^ treatment caused a final longer lasting Ca^2+^ spike (Fig. 5*K*), whereas in other prMC, La^3+^ completely blocked any further spikes (Fig. 5*I*). In all cases, the mathematical model could truthfully recapitulate the experimental findings by changing the parameters *J*_IN_ and *J*_EFF_ at different time points (Fig. 5, *D*, *F*, *H*, *J*, and *L*). Of note during La^3+^ insulations, the width (duration) of Ca^2+^ spikes was wider (longer) both *in vitro* and *in silico*. Moreover, the La^3+^-evoked oscillation block in the presence of extracellular Ca^2+^ ([Ca^2+^]*_o_* ≈ 1 mm) is, according to our model, mostly due to the decreased levels of Ca^2+^ ions present in the different cell compartments; *i.e.* the sum of *c*_cyt_ + *c*_ER_ + *c*_mito_ is smaller than the sum prior to agonist administration.

##### Effect of the Intracellular Buffer Calretinin on Ca^2+^ Oscillations

**FIGURE 6.**
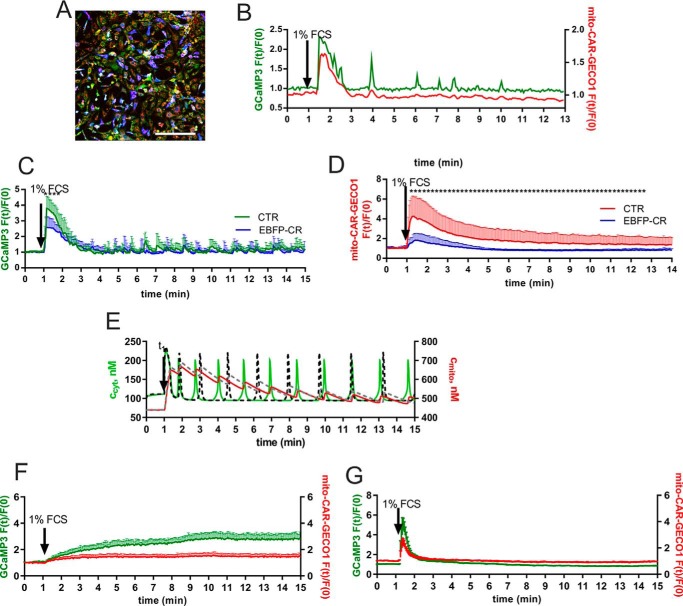
**The effect of increased mobile cytosolic Ca^2+^-buffering capacity resulting from ectopic EBFP-calretinin expression on Ca^2+^ oscillations.** *A*, fluorescence image of cultured prMC expressing the fusion protein EFBP-calretinin (*blue*). The faint *green* and *red* fluorescence signals represent the basal fluorescence intensities of GCaMP3 (cytosolic Ca^2+^ indicator) and mito-CAR-GECO1 (mitochondrial Ca^2+^ indicator), respectively. The faintly fluorescent *non-blue* cells are control cells not expressing EFBP-calretinin. The *scale bar* represents 250 μm. *B*, single cell fluorescence intensity recordings from time lapse videos show a representative Ca^2+^ response in *c*_cyt_ and *c*_mito_ of cells expressing EBFP-calretinin. Oscillations were only observed in cells with faint *blue* fluorescence, *i.e.* relatively low EBFP-calretinin expression levels. Note the relatively small signal in *c*_cyt_ as compared with control cells (*e.g.* in Figs. 2*C* and 4*B*). *C*, Ca^2+^ oscillations were monitored in a mixed population of mito-CAR-GECO1/GCaMP3 prMC (*green* trace) and EBFP-calretinin/mito-CAR-GECO1/GCaMP3 prMC (*black* trace). Averages of *c*_cyt_ values of 10 randomly selected oscillatory prMC each were plotted; data are mean + S.D. Serum-induced maximal amplitudes in *c*_cyt_ were considerably lower in EBFP-calretinin-expressing prMC. *D*, a similar reduction was also seen for *c*_mito_ in oscillatory prMC; data are mean + S.D. from eight cells each. *E*, in the simulation, the known Ca^2+^ binding properties of calretinin were incorporated. The addition of calretinin (0.5 μm) resulted in lower amplitudes of *c*_cyt_, and the mitochondrial uptake (reflected by *c*_mito_) was decreased in the presence of calretinin. *F*, BAPTA-AM preloading (30 μm for 15 min) resulted in a low amplitude plateau response in *c*_cyt_ (*green*) and an even smaller one in *c*_mito_ without Ca^2+^ oscillations. Also the initial serum-induced Ca^2+^ spike was completely abolished. *G*, in EGTA-AM preloaded (30 μm for 15 min) prMC, serum readministration evoked single Ca^2+^ spikes of short duration in *c*_cyt_ (*green*) and *c*_mito_ (*red*). Only a few cells (<1%) showed a second Ca^2+^ transient, but oscillations in *c*_cyt_ were completely blocked by 30 μm EGTA. *F* and *G*, averages of *c*_cyt_ and *c*_mito_ values of 10 randomly selected prMC were plotted; data are mean + S.D. *Asterisks* indicate significance at *p* < 0.05. *Error bars* represent S.D. *CTR*, control.

Based on previous findings that human mesothelioma *in vivo*, mesothelioma cells *in vitro*, and reactive mesothelial cells express calretinin (60, 61), we hypothesized that prMC also might express this protein and that its presence might affect the Ca^2+^ oscillations. However, calretinin protein expression levels in prMC were found to be below the detection limit of our Western blot analysis (8), *i.e.* lower than ∼100 nm and thus unlikely to affect the Ca^2+^ oscillations as the result of the Ca^2+^-buffering capacity of calretinin. In support of this assumption, oscillation patterns (frequency, amplitude, and duration) in prMC from either wild type or calretinin knock-out (CR−/−) mice were indistinguishable (data not shown). However, to mimic the situation of calretinin-expressing reactive mesothelial cells and to investigate the putative role of calretinin in those cells, we overexpressed a fusion protein consisting of EBFP separated from full-length calretinin by a small linker peptide by infection of prMC with the appropriate lentivirus. We estimated in a semiquantitative way by Western blot analyses the expression levels of EBFP-calretinin. The expression level was found to be ∼75 pg of EBFP-calretinin/μg of total protein, leading to an estimated upper concentration of 250 μm calretinin. The EBFP tag on calretinin served as a marker for the distinction of the two populations with or without calretinin (Fig. 6*A*). The percentage of infected cells was usually higher than 90%. The fraction of prMC showing Ca^2+^ oscillations (∼10–20%) was considerably lower than in non-infected cells not expressing calretinin (∼60–70%) and moreover was restricted to cells showing faint blue fluorescence, *i.e.* low EBFP-calretinin expression levels. In the oscillating EBFP-calretinin-expressing prMC, the Ca^2+^ spike amplitudes were smaller, and the half-width of Ca^2+^ spikes (duration) was increased (Fig. 6*B*). The largest effect caused by EBFP-calretinin was the reduction of the amplitude of the first Ca^2+^ spike after serum readministration (Fig. 6*C*); on average it was half the size compared with the situation without calretinin. Likely as the consequence of the reduction in *c*_cyt_, the increase in *c*_mito_ also was clearly diminished (Fig. 6*D*). The frequency of oscillation slightly decreased (∼10–20% reduction in each time segment). Our model simulations incorporating calretinin with the known Ca^2+^ binding characteristics (54) showed similar modifications: a decrease both in the amplitudes of *c*_cyt_ spikes and in the amount of mitochondrial Ca^2+^ uptake (Fig. 6*E*). In our model, an increase in calretinin concentration resulted in an increase of the oscillation frequency, a prediction not supported by our experimental findings. One reason may be that calretinin, in addition to its buffering capacity, might act as a Ca^2+^ sensor in prMC. We had previously shown that calretinin is able to directly modify the activity of a Ca^2+^ channel (60), and direct targets for calretinin implicated in Ca^2+^ transportation might also be present in prMC. As a control to exclude that observed effects were mediated by the EBFP part of the fusion protein, prMC were infected with the lentivirus LV-EBFP2-X leading to the expression of EBFP only. No differences in the Ca^2+^ oscillations patterns were observed between cells expressing EBFP and non-infected control cells (data not shown). Based on the fact that Ca^2+^ oscillations in EBFP-calretinin-expressing cells were limited to those with faint fluorescence, we reasoned that the concentration in these cells was ∼10-fold (*e.g.* 25 μm) lower than the global concentration (250 μm) estimated from Western blot analyses. Thus, we tested whether the commonly used synthetic Ca^2+^ chelators BAPTA and EGTA, which have different properties (*e.g. K_d_*, on-rate constant (*k*_on_), and diffusion coefficient (*D*)) than calretinin, were able to recapitulate the effects of calretinin. In prMC loaded with BAPTA-AM (30 μm), serum readministration evoked a slow and prolonged *c*_cyt_ elevation paralleled by a minute increase in *c*_mito_ (Fig. 6*F*). Most importantly, the initial rise in *c*_cyt_ as also seen in EBFP-calretinin-expressing prMC (Fig. 6*C*) was completely abolished. In contrast, after EGTA-AM loading serum, readministration induced a short spike both in *c*_cyt_ and *c*_mito_ (Fig. 6*E*) followed by a rapid return to essentially baseline levels. No Ca^2+^ oscillations were observed in both cases. This further indicates that the properties of calretinin are clearly distinct from those of either BAPTA or EGTA.

## Discussion

Characteristics of mitochondrial Ca^2+^ transport have not been examined in detail in most cell types. The main reason why we know relatively little about mitochondrial Ca^2+^ handling is because the molecular identity of the channels involved in mitochondrial transport have only recently been discovered (18, 62, 63), and specifically targeted, pH- and ΔΨ-insensitive Ca^2+^ indicators are only currently available (23). Nevertheless, there are few models for Ca^2+^ oscillations where the function of mitochondrial Ca^2+^ uptake has been taken into account (64).

Our experiments affirm previous data that mitochondria, even at the resting state, are able to transport and store Ca^2+^ ions (65). The fast release of the stored Ca^2+^ from the mitochondria due to the decrease/collapse of the membrane potential indicates that the strongly negative ΔΨ ensures a constant Ca^2+^ uptake into the mitochondria. This uptake is in a steady-state equilibrium with the constant Ca^2+^ efflux mediated by the mitochondrial exchangers (66), and the efflux is an electrogenic process (67). The electrochemical proton gradient across the inner mitochondrial is used to remove the excess Ca^2+^ ions (68). Our recordings show that this basal steady-state mitochondrial Ca^2+^ concentration can fluctuate, showing “spontaneous” mitochondrial Ca^2+^ spikes. Most probably this is mediated by an endogenous MCU activator that has not been identified at the molecular level yet. Ca^2+^ transients in *c*_cyt_ were previously reported to evoke an increase in *c*_mito_, activating both cytoplasmic (19) and mitochondrial enzymes (2). Thus, Ca^2+^ transients observed selectively in *c*_mito_ in some prMC (Fig. 2*A*) might allow for the autonomous activation of mitochondrial enzymes. The Ca^2+^ ions causing the mitochondrial spike are likely to originate from the cytosolic compartment; however, our results indicate that the amount of Ca^2+^ ions responsible for the increase in *c*_mito_ was not sufficient to be detected as a decrease in *c*_cyt_. Alternatively, at basal conditions, the equilibrium level of *c*_cyt_ might be regulated by a rather rapid constant exchange of Ca^2+^ ions among the cytosol, the extracellular space, and/or the ER compartment.

The Ca^2+^ oscillation models usually differ in how they simulate the functions of InsP_3_R, the channel that transports Ca^2+^ ions from the ER to the cytosol. The “*c*_cyt_/[InsP_3_]” models (for a review, see Ref. 69) postulate that the InsP_3_R has a binding site for InsP_3_, an activating binding site for Ca^2+^, and an inhibiting binding site for Ca^2+^. In these models, all binding sites are localized on the cytoplasmic side, and the function of InsP_3_ does not depend on *c*_ER_. Binding of Ca^2+^ to the activating site and of InsP_3_ to the InsP_3_ binding site opens the channel, whereas Ca^2+^ binding to the inhibiting site closes the InP_3_R. Moreover, the binding of Ca^2+^ to the inhibiting site occurs rather slowly and with a lower affinity as compared with the activating site, subsequently resulting in oscillations in *c*_cyt_. In these models, the InsP_3_ concentration uniquely determines the oscillation frequency (70). In the “store loading” models (also called “*c*_cyt_/*c*_ER_” models), the function of InsP_3_R depends not only on *c*_cyt_ but also on *c*_ER_. In these models, the Ca^2+^ influx across the plasma membrane plays a critical role in determining the oscillation frequency (8, 71, 72). At a constant [InsP_3_], the duration of the interspike period is determined by the velocity of cellular Ca^2+^ replenishment, which is manifested as a continuous ER loading together with a constant basal *c*_cyt_. The experimentally observable sawtooth wave oscillations in *c*_ER_ during the cytoplasmic baseline spiking oscillations are an important argument in favor of the store loading theory (8). However, the store loading-based models cannot cope with the fact that in some cells the Ca^2+^ oscillations do not depend on Ca^2+^ influx across the plasma membrane. Our experiments and modeling studies revealed that the incorporation of mitochondria as an additional Ca^2+^ source/store in the store loading-based models considerably augments the quality of the simulations. That is, the modeling predictions are more congruent with the experimental findings, which allows for a better mechanistic understanding. The mitochondrial Ca^2+^ transport enables the store loading-based models also to display Ca^2+^ oscillation in the absence of extracellular Ca^2+^.

The simulation of the La^3+^ insulation was previously endeavored by Sneyd *et al.* (73). Although their model does not contain mitochondria and moreover *c*_cyt_ is continuously decreasing during the oscillations, their model reveals important aspects of the Ca^2+^ oscillations, namely their dependence on the total Ca^2+^ load of the cell. In their model, the cell has a high resting Ca^2+^; upon agonist stimulation, the activation of plasma membrane Ca^2+^-ATPases causes a net loss of Ca^2+^ from the cells even though the Ca^2+^ influx is augmented after stimulation (73). A similar phenomenon is also observed in our model; the total cellular Ca^2+^ content (*c*_cyt_ + *c*_ER_ + *c*_mito_) determines the response to the La^3+^ insulation; blocking of the Ca^2+^ influx and efflux results in an oscillation stop that can either occur after a final Ca^2+^ spike or directly after La^3+^ addition, *i.e.* without a change in *c*_cyt_. In contrast to the previous model (73), basal *c*_cyt_ levels during the interspike phase of the oscillations remain constant. This is in line with the experiments carried out by us and others (74).

Shuttling of Ca^2+^ ions between the ER and mitochondria was experimentally demonstrated in the study of Ishii *et al.* (9). They reported that in HeLa cells the cycles of ER/mitochondrion shuttling are repeated until *c*_mito_ has reached the basal level prior to the stimulation. In our study with prMC, we observed Ca^2+^ oscillation even (i) when *c*_mito_ had reached its basal levels or (ii) if *c*_mito_ had been considerably lowered by CCCP administration. One has to keep in mind that CCCP also results in the collapse of the plasma membrane potential (75), which subsequently reduces the plasmalemmal Ca^2+^ influx (76). Thus, one reason for the CCCP-evoked stop in oscillations might be a disturbed Ca^2+^ influx. Moreover, the CCCP-mediated drop in ATP production likely leading to an impairment of the ER Ca^2+^ transport might also contribute to the oscillation arrest (77); *i.e.* the effects of protonophores are not exclusively attributed to the reduced mitochondrial Ca^2+^ uptake as was proposed in earlier studies (9). When CCCP was administered before serum, it caused a Ca^2+^ transient due to the mitochondrial release, which was followed by a period of lower resting *c*_cyt_. A lower *c*_cyt_ is a sign of the reduced Ca^2+^ influx (resting plasmalemmal Ca^2+^ leakage). There was a similar decrease in resting *c*_cyt_ when the extracellular free Ca^2+^ was chelated by EGTA (data not shown).

The Ca^2+^ influx across the plasma membrane is important to sustain the Ca^2+^ oscillations in prMC (78) but not in HeLa cells (9). The different dependence of these cell types on extracellular Ca^2+^ for the oscillations might be the result of differences in the contribution/importance of the various Ca^2+^ shuttling pathways between ER and mitochondria on the one hand and between ER and the extracellular space on the other. Our results indicate that plasmalemmal Ca^2+^ extrusion systems and mitochondrial Ca^2+^ uptake channels compete for the Ca^2+^ ions released from the ER. We hypothesize that in some cells, such as prMC and HEK cells (74), the shuttling between the extracellular space and the ER dominates over the shuttling between mitochondria and the ER. However, in HeLa cells and hepatocytes, the ER/mitochondrion shuttling prevails. This might explain why Ca^2+^ oscillations in some cells are strongly dependent on extracellular Ca^2+^ ions but not in others.

**FIGURE 7.**
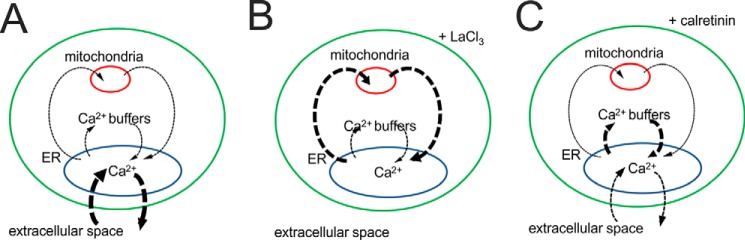
**Contribution of Ca^2+^ signaling toolkit components to serum-induced Ca^2+^ oscillations in prMC.** *A*, in unperturbed (control) prMC *in vitro*, Ca^2+^ oscillations are primarily the result of the interplay between Ca^2+^ from the extracellular space and the ER with some minor contributions of mitochondria. The *arrows* indicate the shuttling of Ca^2+^ ions between the different compartments (the *thicker* the *arrow*, the more important is this pathway). In prMC, expression of calretinin is virtually absent, excluding an important role of this protein with respect to mobile Ca^2+^ buffering. *B*, if cells are subjected to La^3+^ insulation excluding the exchange of Ca^2+^ ions via the plasma membrane, the repetitive Ca^2+^ exchange between the ER and mitochondria allows for the generation of Ca^2+^ oscillations. *C*, the addition of the mobile Ca^2+^ buffer calretinin as observed in reactive mesothelial cells and mesothelioma cells affects Ca^2+^ oscillations. High expression levels (>1 μm in our model) completely block oscillations; lower levels (≈0.5 μm) reduce the amplitude of *c*_cyt_ as well as of *c*_mito_ during Ca^2+^ oscillations; *i.e.* calretinin competes with mitochondria, thus reducing the shuttling of Ca^2+^ ions between the ER and mitochondria.

Another often neglected aspect about “Ca^2+^ shuttling” pathways is the contribution of cytosolic Ca^2+^ buffers present at rather high concentrations in the cytosol of some cell types. They are expected to modulate the Ca^2+^ shuttling among all compartments, extracellular space, ER, and mitochondria, as well as to transiently affect *c*_cyt_ (Fig. 7). A strong interdependence between cytoplasmic Ca^2+^ buffers and mitochondria has been demonstrated before. The expression levels of parvalbumin, a Ca^2+^-buffering protein with slow binding kinetics, and the mitochondrial volume in fast twitch muscle cells and in parvalbumin-expressing neurons are inversely regulated (Ref. 79, and for more details, see Ref. 80). In our study, we observed that overexpression of calretinin modifies Ca^2+^ signals and associated oscillations. It reduces the amount of Ca^2+^ ions shuttling both between the ER and mitochondria and between the ER and the cytoplasm. Our model predicts that at calretinin concentrations >1 μm Ca^2+^ oscillations should be blocked in prMC. This is in apparent contradiction with the experimental results where oscillations still existed in EBFP-calretinin-expressing cells likely expressing levels higher than 1 μm (Fig. 6). However, in our modeling, the Ca^2+^ microdomain was not considered, and Ca^2+^ binding characteristics of calretinin (*e.g. K_d_* and *k*_on_) might be different in the cytosol of prMC than the parameters determined *in vitro* (34). Furthermore, adaptation/compensation mechanisms might be induced in prMC overexpressing calretinin that would still allow for the generation of Ca^2+^ oscillations.

Of relevance, calretinin reduced the mitochondrial Ca^2+^ uptake and Ca^2+^ accumulation. In human malignant mesothelioma, mostly of the epithelioid type, calretinin is overexpressed (81). This might cause changes, *e.g.* a delay or blocking of apoptotic/necrotic processes (78, 82). Thus, the increased calretinin expression in mesothelioma cells and moreover in certain colon cancer (83) and derived cell lines (84) might be correlated or causally linked to the increased resistance of these tumor cells to the apoptotic/necrotic signals either occurring in healthy physiological conditions or resulting from treatment with chemotherapy drugs such as oxaliplatin or 5-fluorouracil (85). In support, colon cancer cells resistant to aurora kinase inhibitors are characterized by higher calretinin expression levels (86). Moreover, down-regulation of calretinin by lentiviral infection induces apoptosis in mesothelioma cell lines *in vitro* via an intrinsic mitochondrion-mediated pathway (87). Also down-regulation of calretinin in colon cancer cells is associated with cell growth arrest and increased apoptosis (88).

## Author Contributions

L. P. designed the study, performed the experiments with simulations, and wrote the paper. W. B. provided assistance, contributed to lentivirus production and cloning (CALB2), and performed qRT-PCR. B. S. secured funding, analyzed data, and wrote the paper.

## Supplementary Material

Supplemental Data

## Footnotes

2 The abbreviations used are:

ER: endoplasmic reticulum
SERCA: sarco/endoplasmic reticulum Ca^2+^-ATPase
*c*_cyt_: cytosolic free Ca^2+^ concentration
*c*_nucl_: nuclear free Ca^2+^ concentration
*c*_ER_: free Ca^2+^ concentration in the ER lumen
*c*_mito_: free Ca^2+^ concentration in the mitochondrial matrix
MCU: mitochondrial calcium uniporter
CCCP: carbonyl cyanide *m*-chlorophenylhydrazone
prMC: primary mouse mesothelial cells
EBFP: enhanced blue fluorescent protein
InsP_3_R: inositol trisphosphate receptor
InsP_3_: inositol trisphosphate
ΔΨ: mitochondrial membrane potential
BAPTA: 1,2-bis(2-aminophenoxy)ethane-*N*,*N*,*N*′,*N*′-tetraacetic acid
AM: acetoxymethyl ester
CR: calretinin
qRT-PCR: quantitative RT-PCR
ROI: region of interest.
