# Supplementary material for: Routes of Ca2+ Shuttling during Ca2+ Oscillations: FOCUS ON THE ROLE OF MITOCHONDRIAL Ca2+ HANDLING AND CYTOSOLIC Ca2+ BUFFERS
Source: J Biol Chem. 2015 Sep 22;290(47):28214–30. doi: 10.1074/jbc.M115.663179 (PMC4653679; doi:10.1074/jbc.M115.663179)
Supplement: Supplemental Data [file supp_290_47_28214__index.html]

Routes of Ca2+ Shuttling during Ca2+ Oscillations; Focus on the Role of Mitochondrial Ca2+ Handling and Cytosolic Ca2+ Buffers — Routes of Ca2+ Shuttling during Ca2+ Oscillations — Ca2+ Shuttling during Ca2+ Oscillations in Mesothelial Cells — Supplemental Data 

# Routes of Ca2+ Shuttling during Ca2+ Oscillations

## Supplemental Data

- Supplementary Excel document (.xlsx, 4.8 MB) - An example for mathematical simulation
